# Taming the Production of Bioluminescent Wood Using the White Rot Fungus Desarmillaria Tabescens

**DOI:** 10.1002/advs.202403215

**Published:** 2024-09-12

**Authors:** Francis W. M. R. Schwarze, Tiago Carvalho, Giacomo Reina, Luiz Garcia Greca, Urs Buenter, Zennat Gholam, Leonard Krupnik, Antonia Neels, Luciano Boesel, Hugh Morris, Markus Heeb, Anja Huch, Gustav Nyström, Giorgia Giovannini

**Affiliations:** ^1^ Laboratory for Cellulose and Wood Materials Empa Lerchenfeldstrasse 5 St. Gallen 9014 Switzerland; ^2^ Laboratory for Particles‐Biology Interactions Empa Lerchenfeldstrasse 5 St. Gallen 9014 Switzerland; ^3^ Center for X‐ray Analytics Empa Lerchenfeldstrasse 5 St. Gallen 9014 Switzerland; ^4^ Giorgia Giovannini Laboratory for Biomimetic Membranes and Textiles Empa Lerchenfeldstrasse 5 St. Gallen 9014 Switzerland; ^5^ Integrated Land Management Department SRUC Barony, Parkgate Dumfries DG1 3NE UK

**Keywords:** bioluminescence, hybrid living materials, selective delignification, wood decay

## Abstract

Although bioluminescence is documented both anecdotally and experimentally, the parameters involved in the production of fungal bioluminescence during wood colonization have not been identified to date. Here, for the first time, this work develops a methodology to produce a hybrid living material by manipulating wood colonization through merging the living fungus Desarmillaria tabescens with nonliving balsa (Ochroma pyramidale) wood to achieve and control the autonomous emission of bioluminescence. The hybrid material with the highest bioluminescence is produced by soaking the wood blocks before co‐cultivating them with the fungus for 3 months. Regardless of the incubation period, the strongest bioluminescence is evident from balsa wood blocks with a moisture content of 700–1200%, highlighting the fundamental role of moisture content for bioluminescence production. Further characterization reveals that D. tabescens preferentially degraded hemicelluloses and lignin in balsa wood. Fourier‐transform infrared spectroscopy reveals a decrease in lignin, while X‐ray diffraction analysis confirms that the cellulose crystalline structure is not altered during the colonization process. This information will enable the design of ad‐hoc synthetic materials that use fungi as tools to maximize bioluminescence production, paving the way for an innovative hybrid material that could find application in the sustainable production of light.

## Graphical abstract

Here, this work describes an efficient method to induce bioluminescence in non‐living balsa wood by treating it with the living fungus Desarmillaria tabescens. The optimized growth conditions maximize colonization of the wood by rhizomorphs. These findings show that the moisture content and the breakdown of lignin are essential factors in the production of bioluminescent wood.

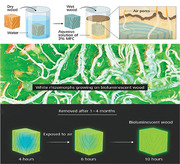

## 1 Introduction

Future advanced materials must have “smart” functional qualities that go beyond what is now possible, like the capacity to self‐heal and respond to environmental cues and changes in state.

From studying nature firsthand, significant attempts have been made to develop biohybrids, which are living/nonliving composite materials that can sustain and regulate the functionality of biological agents.^[^ ^1^ ^]^ Nguyen et al.,^[^ ^2^ ^]^ reviewed early efforts on engineered living materials (ELMs), with a focus on living composite materials that incorporate inorganic components, large‐scale implementation, and production techniques. ELMs are formed of living cells embedded in a dead organic substance, which provide the responsive function to the material. The biohybrid material due to the incorporation of a living component can hence respond to environmental stimuli resulting in functionality.^[^ ^2^ ^]^ ELMs may also consist of hybrid living materials (HLMs), which combine artificially produced materials with living cells that serve as the functional “smart” components.^[^ ^3^ ^]^

The creation of a hybrid system with both living and nonliving elements adds complexity and functionality to typically inorganic/organic materials. It makes it possible to construct composites with self‐maintenance features, resulting in autonomous self‐healing. An example of application is HLM reactivation, concrete fissures repaired by bacteria based on the precipitation of CaCO_3_ through metabolism of bacterial spores in a concrete mixture.^[^ ^4^, ^5^ ^]^ Alongside bacteria, ELMs made of fungal cells offer noteworthy potential due to their functional properties such as self‐assembly,^[^ ^6^ ^]^ self‐healing,^[^ ^7^ ^]^ sensing,^[^ ^8^, ^9^ ^]^ and the production of plant secondary metabolites.^[^ ^10^ ^]^

Bioluminescence from glow producing fungi present in decaying wood could be considered a natural biohybrid. The functionality of bioluminescent wood was confirmed by miners in the 18^th^ century, who used glowing timber props as a guide in replacement of torches.^[^ ^11^, ^12^ ^]^ In 1912, Molisch,^[^ ^13^ ^]^ observed that the light emitted in mines was from mycelial strands (rhizomorphs) produced by honey fungus (Armillaria sp). This observation was scientifically proven by Guyot.^[^ ^14^ ^]^ Bioluminescent wood, called foxfire, is a phenomenon in nature that is caused by white rot fungi in decaying wood in the dark. The fox in foxfire is thought to derive from the old French word faux, meaning false, in reference to the bioluminescent glow mimicking fire. Both Aristotle (384–322 BC) and Pliny the Elder (23–79 AD) reported the glow in ancient times.^[^ ^15^, ^16^ ^]^

Bioluminescence in fungi occurs across phylogenetic lineages, including the genus Armillaria.^[^ ^17^, ^18^ ^]^ To date, 40 species of Armillaria that degrade lignin in wood have been described.^[^ ^19^ ^]^ Most studies on the bioluminescence of fungi have been conducted enzymatically from cold and hot water extracts,^[^ ^20^ ^]^ fruiting bodies,^[^ ^21^, ^22^ ^]^ mycelium on artificial growth media,^[^ ^23^, ^24^ ^]^ or from naturally infected wood samples collected in the field.^[^ ^25^ ^]^ The degradation of lignin by Armillaria spp. results in the accumulation of phenylpropanoids. Among these, caffeic acid, is further metabolized into hispidin via the caffeic acid cycle.^[^ ^26^ ^]^ Afterward, this cycle continues, and hispidin is then transformed into 3‐hydroxyhispidin, also known as luciferin.^[^ ^27^ ^]^ In the presence of oxygen, the enzyme luciferase induces the oxidation of luciferin into a high‐energy intermediate, which is then metabolized forming pyruvate (required to produce energy), and caffeic acid, releasing energy in the form of bioluminescence, emitting a bluish‐green glow.^[^ ^28^, ^29^ ^]^ This series of enzymatic reactions that are responsible for fungal bioluminescence are highly affected by cultivation factors such as the presence of oxygen,^[^ ^26^ ^]^ and the lignolytic activity of the fungus.^[^ ^30^ ^]^

Although, bioluminescence from Armillaria spp. has been documented both anecdotally and experimentally,^[^ ^27^, ^28^ ^]^ to our knowledge, the chemical reaction has not been artificially induced in wood through manipulation of the conditions for fungi to glow. The main reason for this is that the balance between the choice of fungal species, wood species, its moisture content and the environmental conditions required to produce bioluminescent wood is very challenging. Investigating the production of bioluminescent wood, we hypothesized that using the white rot fungus Desarmillaria tabescencs will lead to bioluminescence, because of its ability to colonize and degrade lignin in wood with a very high moisture content. After dehydrating the wood, facilitating its exposure to oxygen, the caffeic acid cycle will be induced. As described previously, this will lead to the oxidation of luciferin, resulting in visible bioluminescent light emission.

Taking the above factors into account, in this study, we developed a hybrid living material using the white rot fungus D. tabescens and balsa wood (Ochroma pyramidale) by fusing the living fungus with the nonliving wood to produce a bioluminescent material, with the aim to accomplish multifunctionality, to help address societal challenges.

## 2 Results and Discussion

**Figure 1:**
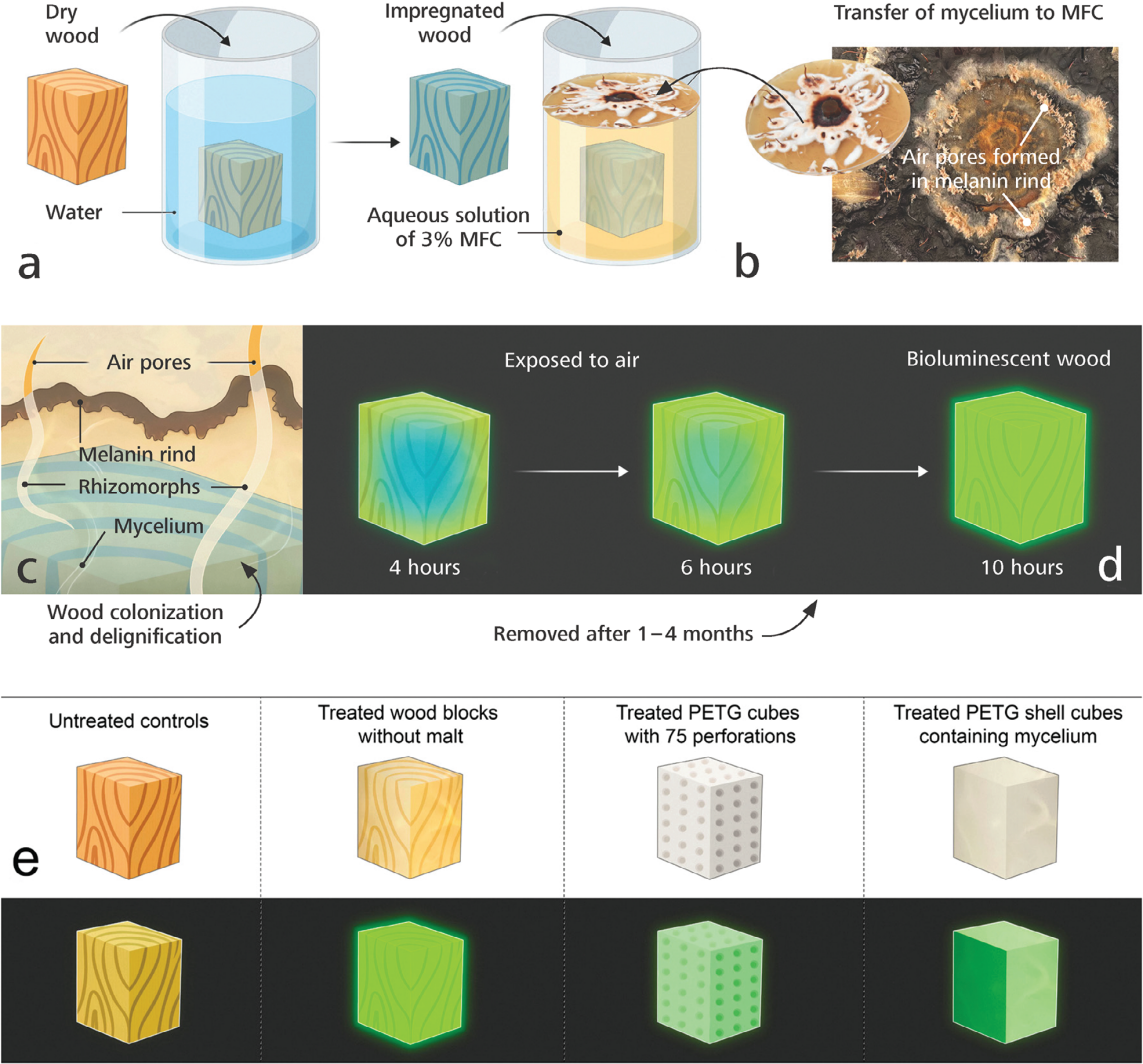
Schematic demonstrating the experimental set up for producing bioluminescent wood. a) Balsa wood blocks were impregnated in a vacuum‐pressure chamber thereby absorbing significant amounts of water into its structure and then autoclaved. b) For cultivation of D. tabescens, an aqueous growth media containing 3% microfibrillated cellulose (MFC) was used. Living mycelium was transferred to the growth media and air pores developed in tufts within the melanized rind that covered the surface of MFC. c) Balsa wood blocks submerged in MFC were colonized by rhizomorphs and mycelium of D. tabescens. d) After 1–4 months incubation, wood blocks were removed, cleaned and exposed to air in the dark. As excess water evaporated, emission of bioluminescence increased over time. e) A comparison of bioluminescence from untreated controls, treated wood and non‐lignified materials, that is, transparent PETG cubes or cube shells. The strongest bioluminescence was recorded from treated wood blocks, whereas in the absence of lignin in PETG cubes or cube shells emission of bioluminescence was weak.

A summary of the steps for producing bioluminescent wood are summarized in **Figure** **1**A–D. Balsa, a light low‐density wood, absorbed significant amounts of water (>1000% (w/w)) into its structure (Table S1, Supporting Information). The high water uptake can be explained by the low mean density (0.094 ± 0.012 g cm^−3^) of the wood samples (n = 120) used in this study. The optimum moisture content for the emission of bioluminescence after 3 months incubation was 814 ± 44.8% (Table S1, Supporting Information). As the moisture is an important factor for an extended bioluminescence, it is questionable whether high density wood species can absorb enough water to glow strongly.

Under low oxygen conditions, wood decay fungi and insects cannot colonize wood, giving D. tabescens a competitive advantage. Thus, decay by closely related Armillaria spp. has been reported to occur in wet storage, even when irrigation levels were high. Thus, in saturated sapwood they form air pores enabling oxygen to diffuse into the tissue for wood decay to follow.^[^ ^31^, ^32^ ^]^ The latter very wet conditions are also an important prerequisite for selective delignification by D. tabescens.^[^ ^32^, ^33^ ^]^

**Figure 2:**
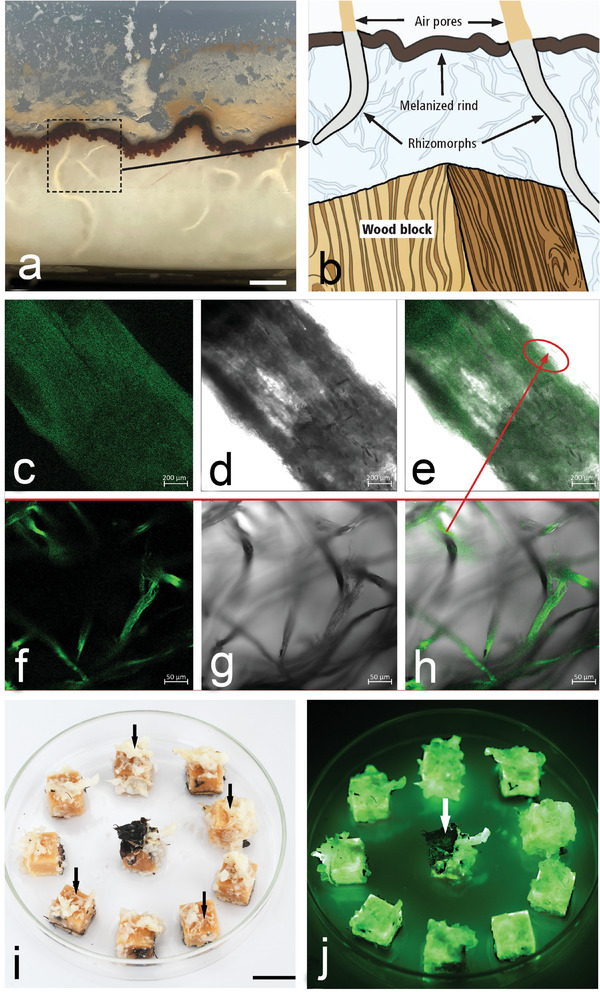
Incubation of wood blocks in 3% MFC with D. tabescens for producing bioluminescent wood. a) Non‐melanized rhizomorphs (arrow) colonize MFC and the submerged wood blocks. Scale bar = 1 cm. b) Illustration showing wood blocks submerged in MFC and the connection of rhizomorphs and air pores (arrows). At the substrate‐air interface, MFC is covered by a melanized rind (arrow). c‐h) Merged transmission and fluorescence images obtained by confocal microscopy. Fluorescence of rhizomorphs c–e) and peripheral hyphae f‐h) that formed a loose network, ≈20–30 µm thick, surrounding rhizomorphs. Left – right: Fluorescence, transmittance, merged. i) Balsa wood blocks incubated for 3 months with D. tabescens and exposed to light. Note abundant rhizomorphs (arrows) of D. tabescens have strongly colonized Balsa wood blocks (scale bar = 1.5 cm). j) Wood blocks in i) exposed to air in the dark showing strong bioluminescence induced by D. tabescens. Note dark melanin rind (arrow) absorbing bioluminescence emitted from wood (scale bar = 1.0 cm).

Balsa wood submerged in MFC,^[^ ^34^ ^]^ were rapidly colonized by rhizomorphs (Ø 0.2–1 cm) and mycelia (Ø 2–5 µm). Only few rhizomorphs were observed after 4 weeks incubation, but after 2‐, 3‐ and 4‐months their abundance increased rapidly (**Figure** **2**a). On the surface of the substrate, D. tabescens formed a thin layer (0.5 cm) of melanin (Figure 2b). Rhizomorphs within the substrate remained white and were connected with thin air pores that developed in the melanin layer in the form of tufts. The porous structures seen at the substrate‐air interface are like the intricate aerating system of A. luteobubalina, which works in gas exchange rhizomorphs and fosters development in low‐oxygen settings by oxygen diffusion.^[^ ^35^ ^]^ This complex, sophisticated aeration system prevents waterlogging and combats desiccation, which may help to explain why some Armillaria spp. and the closely related D. tabescens successfully colonize wood in wet conditions. Fungal melanin is hydrophobic, has antimicrobial properties, and protects rhizomorphs against desiccation and contaminants.^[^ ^36^ ^]^ It also helps to maintain elevated wood moisture content within the substrate.^[^ ^37^ ^]^ The cultivation conditions described above also allow the standardized production of bioluminescent wood. Rhizomorphs and mycelia growing on wood submerged in MFC had a strong bioluminescence when viewed under the confocal microscope (Figure 2c–h). Balsa wood samples strongly colonized by rhizomorphs and mycelium of D. tabescens also emitted a strong bioluminescence (Figure 2i,j). As D. tabescens does not produce melanized rhizomorphs in Pure culture (Figure S1, Supporting Information) or during wood colonization,^[^ ^38^ ^]^ emission of bioluminescence was not impaired. However, due to the partial melanization of the surface of some incubated wood blocks once exposed to air, the bioluminescence was blocked (arrows in Figure 2i,j).

### 2.1 Relation between Bioluminescence, Incubation Time and Moisture Content

**Figure 3:**
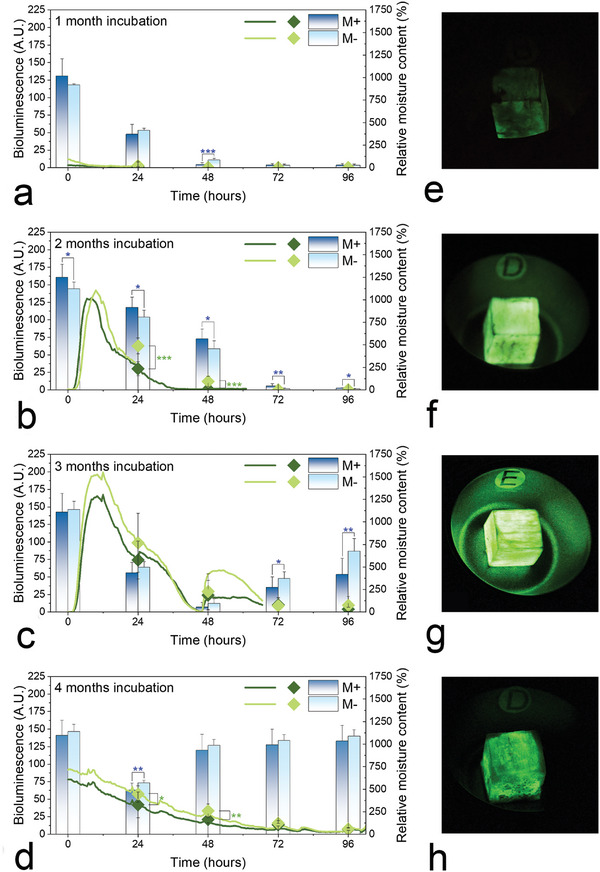
a–d) Relationship between bioluminescence and moisture content of wood blocks incubated for 1, 2, 3, and 4 months over 96 h in a dark room at RT. The bioluminescence measured from images taken each 30 min, using ImageJ, are plotted as lines (dark green for with malt [M+], and light green for without malt [M‐]). The bioluminescence measured each 24 h using a fluorimeter are plotted as diamonds (♦, dark green for M+, and light green for M‐). The relative moisture contents recorded every 24 h are plotted as bars (dark blue for M+, and light blue for M‐). The results are expressed as mean ± SD (n = 10). Levels of significance were set at probabilities of *p < 0.05, **p < 0.01, and ***p < 0.001 and determined by one‐way ANOVA (green* for bioluminescence and blue* for relative moisture content). e‐h) Balsa wood blocks incubated for 1, 2, 3, and 4 months with D. tabescens, after removal of rhizomorphs and mycelium, showing weak e,h) and strong bioluminescence f–g) emission after 10 h exposure to air in the dark, illuminating letters “D” after 2 months and “E” after 3 months.

After removal from MFC, the surface mycelium and rhizomorphs were removed with a brush. Water saturated balsa wood blocks colonized by D. tabescens, did not initially emit bioluminescence in the dark at room temperature. However, after 4 h of exposure to air, loss of moisture from the wood surface due to dehydration via evaporation, resulted in the emission of a greenish light (Figure S3, Supporting Information), as revealed by images obtained every 30 min, and measured with a fluorimeter every 24 h (**Figure** **3**a–d). Bioluminescence generates light signals within visible wavelength (λ_max_ = 560 nm). A marked relationship between incubation period and moisture content of wood was evident. During dehydration, the weakest bioluminescence was recorded from wood blocks after 1 month, whereas the strongest was recorded after 3 months of incubation (Figure 3a,c). Regardless of the incubation period, the strongest bioluminescence was evident from wood blocks with a relative moisture content of 700–1200% (Table S1, Supporting Information).

In wood blocks incubated for 1 and 4 months, respectively, the strongest bioluminescence was observed using a fluorimeter after 30 min of exposure to air, while the glow occurred after 8–12 h of exposure to air in wood incubated for 2 and 3 months (Figure 3a,d). After the latter incubation periods the letters D and E are clearly illuminated in the dark (Figure 3e–h). The very low bioluminescence after a 1‐month incubation appeared to be related to the weak colonization of wood blocks by rhizomorphs, low lignin degradation and the subsequently low production of luciferin (Figure 3a,e). Thus, illumination of the letter in the dark was not apparent. After 4‐months of incubation, the low bioluminescence measured was owing to wood by then being extensively delignified, resulting in a reduction of hispidin synthesis and ultimately luciferin. Significant differences in bioluminescence of wood blocks impregnated with and without 4% malt extract were only measured after 2 months of incubation and air exposure of 24‐h. For all other measurements, emission of bioluminescence was stronger from wood blocks not impregnated with malt (Figure 3a,d).

To assess the influence of hydration on bioluminescence after a 3 month incubation period, and 24‐h air exposure, 2.5 mL of sterile water was added to each wood block. Hydration had a positive effect on the emission of bioluminescence (Figure 3c and Movie S1, Supporting Information). After 4 months of incubation the same amount of H_2_O was added to wood blocks every 12 h. In both cases hydration resulted in a weak‐to‐strong increase in bioluminescence emission (Figure 3d).

### 2.2 Wood Chemical and Physical Structure

**Figure 4:**
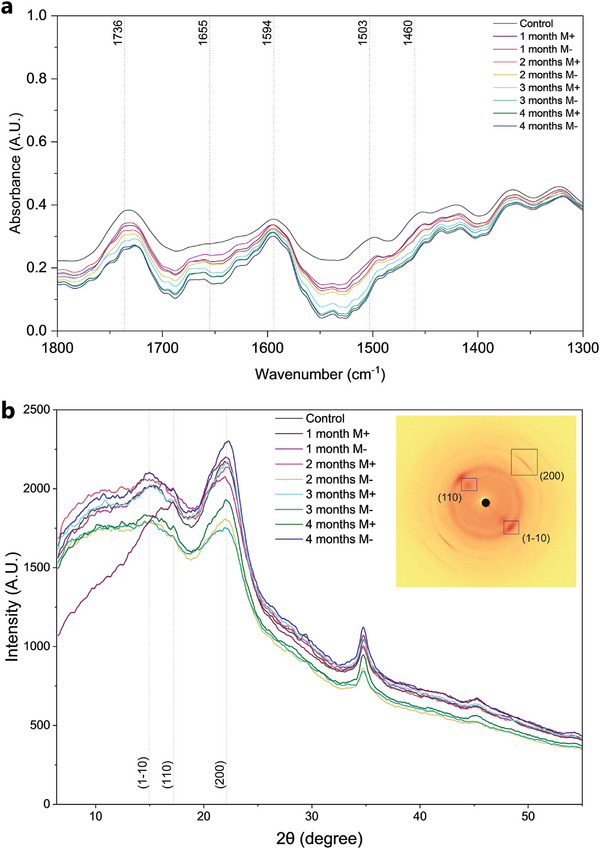
a) ATR‐FTIR showing a decrease and change in the relative intensities of the characteristic lignin bands at 1460 cm^−1^, and hemicellulose fraction, as evidenced by a decline in the relative intensities of bands at 1736 cm^−1^. The complete FTIR spectrum is reported in Figure S4, Supporting Information. b) XRD patterns of untreated controls and treated wood blocks after 1–4 months. Insert 2D detector image of untreated wood showing the characteristic diffraction patterns of the Iβ phase of cellulose.

In nature, many white rot fungi selectively degrade lignin.^[^ ^39^, ^40^, ^41^ ^]^ This phenomenon was similarly observed in our samples by using light microscopy and selective dyes for cellulose (Astra blue) and lignin (safranin, red), respectively (Srebotnik and Messner, 1994).^[^ ^42^ ^]^ In Figure S3, Supporting Information, it is revealed that in the presence of lignin the cell walls in the control samples were strongly stained red as they have the highest lignin content. Then, after 1 month incubation with D. tabescens, in the absence of lignin due to delignification, Astra‐blue stains cellulose in the delignified cell walls blue. After 4 months incubation, in the absence of lignin, all of the cell walls appear blue. Furthermore, applying ATR‐FTIR, we showed that D. tabescens preferentially degraded hemicelluloses and lignin in balsa wood. (**Figures** **4**a and S4, Supporting Information), by a decrease in the relative intensities of their characteristic bands at 1736 and 1460 cm^−1^, respectively.^[^ ^43^ ^]^ Although the appropriate quantification of the wood components after treatment is influenced by the presence of organic compounds derived from the fungi itself, by analyzing the FTIR band areas from Figure 4a, through numerical analysis, it was possible to relatively quantify the lignin and hemicellulose contents. By using this approach, it was possible to observe that the lignin content decreased from 12.25% to 5.48% and 5.89% (M‐ and M+, respectively), and the hemicellulose content decreased from 27.48% to 20.60% and 17.43% (M‐ and M+, respectively, (Table S2, Supporting Information).

Non‐structural carbohydrates are among the more easily accessible to breakdown by fungi.^[^ ^33^ ^]^ In our study, wood blocks impregnated in MFC with 4% malt extract had a higher carbon‐to‐nitrogen (C/N) ratio. It is widely known that a high C/N ratio makes it easier for white‐rot fungi to degrade polysaccharides.^[^ ^44^ ^]^ Therefore, wood blocks without malt have a lower C/N ratio, resulting in a range of white rot fungi to cause selective delignification.^[^ ^45^ ^]^ The effect of malt in wood degradation was supported by our FTIR analysis. We observed a 17.17 ± 80.98% increase of the average lignin consumption, and a 16.00 ± 12.55% increase of the average hemicellulose consumption, when malt was not added to the culture medium (Table S2, Supporting Information). This slight increase in lignin degradation in samples without malt, was reflected by the bioluminescence output, as previously shown in Figure 3a–d, especially after 3 months incubation with D. tabescens, both after 10 h of exposure to the atmosphere, when the excess water was evaporated, and after 48 h, when the wood samples were rehydrated (Figure 3c).

XRD studies on untreated and treated wood with D. tabescens were performed after different incubation periods (Figure 4b). The broad diffraction peaks at 14.9° (1–10), 17.1° (110), and 22.2°. (200), observed for all samples, are associated with the characteristic diffraction peaks of wood.^[^ ^46^, ^47^ ^]^ These diffraction peaks match well with the crystallographic data of the cellulose I_β_ phase (Figure S5, Supporting Information).^[^ ^48^ ^]^ The position of the identified cellulose I_β_ peaks does not change during the different incubation periods, indicating that cellulose Iβ crystalline structure of the decayed wood was not altered during the degradation process. Diffraction peaks of lignin (20.3° and 22.9°),^[^ ^49^ ^]^ could not be determined due to overlapping peaks of the cellulose I_β_ phase. However, considering the mass losses and the low variation in cellulose degradation, the data supports that D. tabescens preferentially degrades lignin and hemicellulose.

In contrast to most hardwood species, the density (≈0.06 to 0.38 g cm^−1^), and lignin content (25%) of balsa wood is very low.^[^ ^50^ ^]^ After 1 month, incubated wood blocks showed no mass loss, but after 2 months, mass loss was significantly higher with a calculated weight percent decrease of 11.0% and 6.5%, for the ones incubated without malt and with malt, respectively (Figure S6, Supporting Information). Malt on the wood surface is more easily accessible and metabolized by fungi, resulting in a lower need to gain nutrients. After 3 and 4 months incubation periods, a slight mass gain was recorded (Figure S6, Supporting Information), although cell wall components were evidently degraded, as previously shown. This result might be explained by the wood colonization by D. tabescens, naturally increasing the final mass of this HLM.

**Figure 5:**
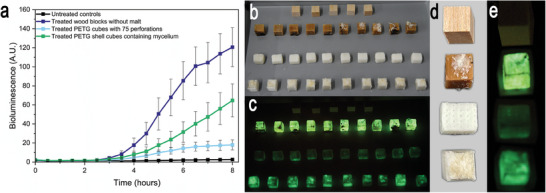
a) Bioluminescence emission by lignified and non‐lignified material colonized by D. tabescens after 2 months incubation. Untreated wood blocks, incubated wood blocks (M‐), incubated transparent PETG cubes with 75 perforations (Ø 0.9 mm) and PETG shells filed with MFC colonized by rhizomorphs and mycelium of D. tabescens (bottom row). b) Materials after 2 months incubation with D. tabescens. c) Bioluminescence of the above materials after 8 h exposure to air in the dark. d) From left – right untreated wood blocks, incubated wood blocks (M‐), incubated transparent PETG cubes and PETG shells filed with MFC, rhizomorphs and mycelium. e) Specimens in d) viewed in the dark emitting bioluminescence. With the exception of controls (n = 5), the results are expressed as mean ± SD (n = 10). Levels of significance were set at probabilities of *p < 0.05, **p < 0.01, and ***p < 0.001 and determined by one‐way ANOVA.

To confirm the important role of the chemical and physical properties of wood in determining the bioluminescence production, we tested the difference between lignified and non‐lignified material (i.e., Polyethylene terephthalate glycol – PETG) colonized by D. tabescens (Figure 1e). The study clearly showed that the emission of bioluminescence from balsa wood was significantly stronger than from the transparent PETG cubes or cube shells (**Figure** **5**). According to calculations, minimal energy is required to produce light, and bioluminescent fungi may be releasing light with little or no heat as an energy by‐product of oxidation reactions mediated by enzymes.^[^ ^51^ ^]^ For example, a relationship between bioluminescence and lignin degradation has been suggested, where white rot fungi may act to detoxify peroxides that are formed during lignolysis.^[^ ^52^, ^53^ ^]^ The findings on both lignified and non‐lignified material incubated with D. tabescens support the hypothesis that fungal bioluminescence is most likely a by‐product of antioxidative metabolism during lignin degradation.^[^ ^52^, ^53^ ^]^ Additionally, bioluminescence might offer antioxidant defense against the harmful effects of reactive oxygen species (ROS), which are primarily produced by mitochondria during respiration.^[^ ^54^ ^]^ We are currently working on a different approach to confirm the influence of the wood properties with the bioluminescence production.

## 3 Conclusions

We have demonstrated an approach to produce a HLM with both living and nonliving elements by manipulating wood colonization by the white rot fungus D. tabescens and by incorporating the living fungus with the nonliving balsa wood to achieve targeted multi‐functionality through the autonomous emission of bioluminescence, also known as foxfire. We have shown that with knowledge of the wood substrate, its moisture content and by manipulating the environmental conditions for wood delignification by the fungus, we made it possible to trigger and standardize emission of bioluminescence. Unlocking the mechanisms behind fungal bioluminescence could provide an electricity free light‐source for the future with a low energy requirement. A battery‐free or plug‐free sustainable light source based on bioluminescent fungi would be beneficial for the environment and help us meet net zero targets in line with the IPCC synthesis report.^[^ ^55^ ^]^ Utilizing bioluminescence to illuminate our homes and communities would result in energy savings and a reduction in CO_2_ emissions. The alternative light source would also reduce nighttime light pollution, a global concern in large cities. For example, bioluminescent wood was used to illuminate the compass and depth gauge of the first submarine, the Turtle, at Benjamin Franklin's suggestion.^[^ ^56^ ^]^

Our studies show that the moisture content of wood, presence of oxygen, amount of lignin and malt as energy source, all strongly influence the intensity of bioluminescence. To make bioluminescence suitable as a green light source, it is fundamental to enhance its intensity and prolong its lifetime. Our current research activities are focused on overcoming these limitations and developing new methods to prolong bioluminescence production. We believe that further studies involving the control of environmental conditions and the selection of specific wood species are key factors in achieving longer and more stable bioluminescence in wood for research, industry, and design.

## 4 Experimental Section

### Comparison of Bioluminescence in Pure Culture

Armillaria cepistipes Velen., Empa 655 and Desarmillaria tabescens (Scop.) R.A. Koch & Aime, Empa 749 (GenBank Accession No. OR135363) were cultivated in Pure cultures on 4% MEA. After 4 weeks incubation photos were taken in the dark from the top (after removal of the lid) and bottom side of plastic Petri dishes at room temperature (22 °C) with a NIKON D850 camera (Japan), mounted on a tripod with a shutter speed F/5, focal length of 40 mm, maximum glare of 4.4 mm, and a 30 s exposure. For these studies, D. tabescens, in contrast to A. cepistipes, was selected as the rhizomorphs of the former do not become melanized (Figure S1, Supporting Information) even after prolonged incubation periods. This is important as melanin acts as a shielding that reduces the bioluminescence emission of rhizomorphs in Pure culture and colonized wood. For cultivation of D. tabescens, this work used an aqueous growth media containing 3% MFC. As shown in Figure 2 balsa wood blocks and non‐lignified materials, that is, transparent PETG cubes or cube shells were impregnated in a vacuum‐pressure chamber with water (with (M+) or without (M‐) 4% malt extract), and then autoclaved.

### Incubation of Balsa Wood Blocks with D. Tabescens

For incubation of wood blocks this work used an aqueous suspension of 3% (w/v) microfibrillated cellulose (MFC) supplied by Weidmann Holding AG, Rapperswil, Switzerland. Before incubation, the wood blocks were autoclaved at 121 °C for 20 min in plastic containers (WEZ, Oberentfelden, Switzerland), with dimensions of 28L × 17 W × 10H cm containing 400 g of aqueous 3% (w/v) MFC and 4% malt. Incubation was carried out using one piece of mycelium (Ø 45 mm) taken from 14‐d‐old pure cultures of D. tabescens. The boxes were incubated in a random array at 22 °C and 75% RH for 1, 2, 3, and 4 months. After incubation, the wood blocks were removed from the boxes, cleaned with a brush and the wet mass measured in g and then converted into %. Loss in moisture content of wood blocks during drying and analysis of bioluminescence was measured every 24 h for 5 days. The mass variation (Δm (%), of each wood block was calculated considering the initial dry mass (W_0_), and the dry mass after incubation (W_1_), according to Equation1:

(1) $$\Delta m=\frac{W_{1}-W_{0}}{W_{0}}\times 100$$

### Adjustment of the Wood Moisture Content

Most scientist working with wood and decay fungi, oven dried their wood and then exposed the wood with a very low moisture content (<20–30%) to the fungus. However, decay by closely related Armillaria spp. was reported to occur in wet storage, even when irrigation levels were high. Wood blocks from balsa (Ochroma pyramidale [Cav. ex Lam.] Urb.) with dimensions of 15 mm (longitudinal) × 15 mm (tangential) × 15 mm (radial) (3.375 cm^3^) were prepared. The mean density (ρ) of control samples (n = 120) was 0.094 ± 0.012 g cm^−3^. A total of 100 wood blocks were prepared for fungal incubation, comprising 10 wood blocks for each of the two treatment and four incubation periods (1, 2, 3, and 4 months). 20 wood blocks were used as controls. Before water uptake measurements, wood blocks were kiln dried at 103 °C for 24 h, then cooled in a desiccator, weighed and the initial dry mass (in g) recorded before incubation. Water retention (in g) of wood blocks was measured, after immersing 50 wood blocks in distilled water and 50 wood blocks in distilled water with 4% malt extract at reduced pressure (700 Pa) for 20 min in a vacuum desiccator according to EN 113. After returning the vacuum tank to air pressure, the wood blocks were submerged in water for a further 120 min before being reweighed.

### Bioluminescence Imaging and Assessment

Wood blocks were placed into a labelled (A‐J) 20‐cup Teflon tray, in a dark room at room temperature (RT). Photos were taken at room temperature (22 °C) in the dark with a NIKON D850 camera (Japan), mounted on a tripod with a shutter speed F/5, focal length of 40 mm, maximum glare of 4.4 mm, and a 30 s exposure every 30 min for 5 d. Each photo was examined using the image processing software ImageJ, to assess the variation of bioluminescence for each wood block over time. The wood blocks were then removed and kiln‐dried at 103 °C for 24 h and then cooled in a desiccator and the final dry mass (in g) recorded. Bioluminescence of wood blocks was measured using a fluorimeter (Horiba FluoroMax, Japan). The instrument setup was adjusted aiming to minimize the effect of bioluminescence. In particular, the sample stage was adjusted by 90 °C so that the wood blocks not exposed to excitation light. The excitation wavelength was set to 280 nm using a cut‐off filter (305 nm, 2 mm, 50 × 50 mm, Reichmann Feinoptik). The emission spectra was recorded between 400 and 600 nm. The peak bioluminescence of incubated wood was observed at 530 nm.

### Light Microscopy

For light microscopy of untreated and delignified wood, samples of ≈ 20 × 5 × 5 mm were cut from the balsa wood blocks. The samples, with transverse faces exposed for examination, were fixed in 2% glutaraldehyde buffered at pH 7.2–7.4, dehydrated with acetone and then infiltrated with a methacrylate medium which was subsequently polymerized at 50 °C. The embedded samples were sectioned at ≈ 20 and 30 µm, using a rotary microtome (Leica 2040 Supercut) fitted with a steel knife. The sections were stained for 12 h in Safranin‐O and then counter‐stained for 30 min in Astra Blue.^[^ ^42^ ^]^ Color micrographs were taken with a Leica DM 4000 microscope using a digital camera and LAS software version 4.13.0.

### Attenuated Total Reflectance–Fourier Transform Infrared Spectroscopy

ATR‐FTIR spectroscopy was performed on thin fragments samples using a Bruker Tensor 27 spectrometer (USA) over a scan range of 4000 to 400 cm^−1^, to detect changes in the chemical composition of treated wood and untreated controls. The spectra was normalized (min/max) and the baseline corrected using OPUS software (Germany).

### X‐ray Diffraction Patterns of Non‐treated and Decayed Wood

XRD measurements were performed using a Stoe IPDS‐II instrument equipped with graphite‐monochromatized Mo‐K_α_ radiation (λ = 0.71073 Å, 40 mA, 50 kV, beam diameter of 0.5 mm) and an image plate detector. The wood samples were fixated onto a rotating sample holder and measured in transmission mode for 30 min with a rotation speed of 10 rpm. The azimuthal data integration of the 2D diffraction pattern resulted in a 1D diffraction profile. XRD data were analyzed by manual identification of the diffraction peaks of the wood samples using the Highscore Plus software suite.

### Bioluminescence of Non‐Lignified Material

For comparative studies of the bioluminescence in wood and non‐wood blocks, 10 cubes and 10 cubical shells made of the transparent polyethylene terephthalate glycol (PETG), with dimensions of 15 mm (longitudinal) × 15 mm (tangential) × 15 mm (radial), were prepared with a Prusa i3 mk3s + Multi Material 2S Kit (MMU2S) 3D printer. The cubes were made with 25 boreholes (Ø 0.9 mm) aligned in the longitudinal, radial and tangential direction. In total, each cube had 75 boreholes. The wood blocks, and cubes were incubated as described above for 2 months with D. tabescens. Each cubical shells (1 mm thick) were filled with 2.5 g of rhizomorphs that had also been incubated in 3% MFC with wood blocks for 2 months. The bioluminescence of all samples were measured as described above.

### Statistical Analysis

The results were expressed as mean ± SD of at least three independent sets of measurements. Statistical analysis was completed using a one‐way analysis of variance (ANOVA), with the level of significance set at probabilities of *p < 0.05, **p < 0.01, ***p < 0.001, analyzed with Origin 2022 software (OriginLab Corp., USA).

## Conflict of Interest

The authors declare no conflict of interest.

## Supporting information

Supporting Information

Supplemental Movie 1

## Data Availability

The data that support the findings of this study are available from the corresponding author upon reasonable request.
