# Supplementary material for: Taming the Production of Bioluminescent Wood Using the White Rot Fungus Desarmillaria Tabescens
Source: Adv Sci (Weinh). 2024 Sep 12;11(44):2403215. doi: 10.1002/advs.202403215 (PMC11600283; doi:10.1002/advs.202403215)
Supplement: Supplementary file 1 — Supporting Information [file ADVS-11-2403215-s002.docx]

Supporting Information

Taming the production of bioluminescent wood using the white rot fungus Desarmillaria tabescens

Francis W.M.R. Schwarze,* Tiago Carvalho, Giacomo Reina, Luiz Garcia Greca, Urs Buenter, Zennat Gholam, Leonard Krupnik, Antonia Neels, Luciano Boesel, Hugh Morris, Markus Heeb, Gustav Nyström, Giorgia Giovannini

**Table S 1.** Showing the maximum bioluminescence over time measured for each wood block, treatment (with and without malt (M+ and M-, respectively)), and corresponding incubation period. The maximum bioluminescence values were measured every 30 min using the maximum value from the entire surface of transverse sections (15 x 15mm) of incubated wood blocks with the processing software ImageJ. The time of maximum bioluminescence, was defined as the time when the image with the highest bioluminescence was measured. The optimum wood moisture content for emission of bioluminescence was calculated by fitting the measured moisture content values into a cubic polynomial and by using the time of maximum bioluminescence as *x* coordinate. The results are expressed as mean ± SD (n ≥ 6).

|  |  | Maximum bioluminescence (A.U.) | | Time at maximum  Bioluminescence (h) | | Optimum wood moisture content (%) | |
| --- | --- | --- | --- | --- | --- | --- | --- |
| 1 month | M+ | 4 ± | 4.6 | 0.8 ± | 1.4 | 991.7 ± | 49.8 |
|  | M- | 12.2 ± | 7.6 | 0.4 ± | 0.2 | 912.4 ± | 6.7 |
| 2 months | M+ | 132.8 ± | 21.8 | 7.3 ± | 0.8 | 1188.7 ± | 7.1 |
|  | M- | 143.8 ± | 12.7 | 9.2 ± | 0.6 | 1042.1 ± | 6.4 |
| 3 months | M+ | 171.5 ± | 51.3 | 10.8 ± | 1.5 | 770.6 ± | 42.8 |
|  | M- | 203.3 ± | 28.3 | 11 ± | 1.6 | 814 ± | 44.8 |
| 4 months | M+ | 80 ± | 20.5 | 1.6 ± | 2.4 | 1055.1 ± | 59.8 |
|  | M- | 95.5 ± | 21.1 | 4.3 ± | 3.6 | 1035 ± | 84.6 |

**
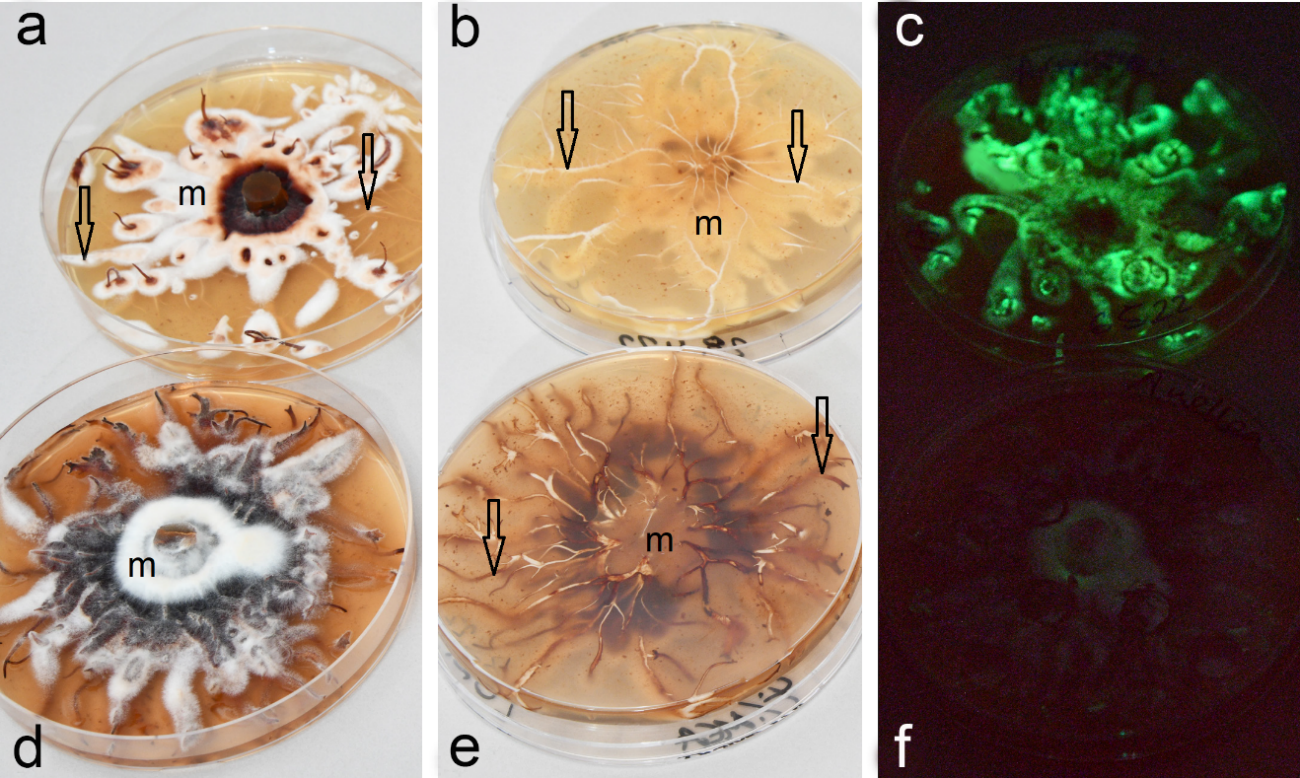
**

**Figure S 1**. a-c) Desarmillaria tabescens cultivated on 4% MEA in Petri dishes. a) Mostly white mycelium (m) and rhizomorphs (arrows) are apparent on the top a) and bottom side b) of the growth media. c) In the dark, mycelium and rhizomorphs of D. tabescens emit a strong bioluminescence. d-e) Armillaria cepistipes cultivated on 4% MEA in Petri dishes. d-e) Mostly dark, melanized mycelium (m) and rhizomorphs (arrows) are apparent on the top d) and bottom side e) of the growth media. f) In the dark melanin-shielding of bioluminescence by melanised rhizomorphs is apparent and the culture appears mostly dark. Only the mycelium in the center of the Petri dish shows a very weak bioluminescence.

**
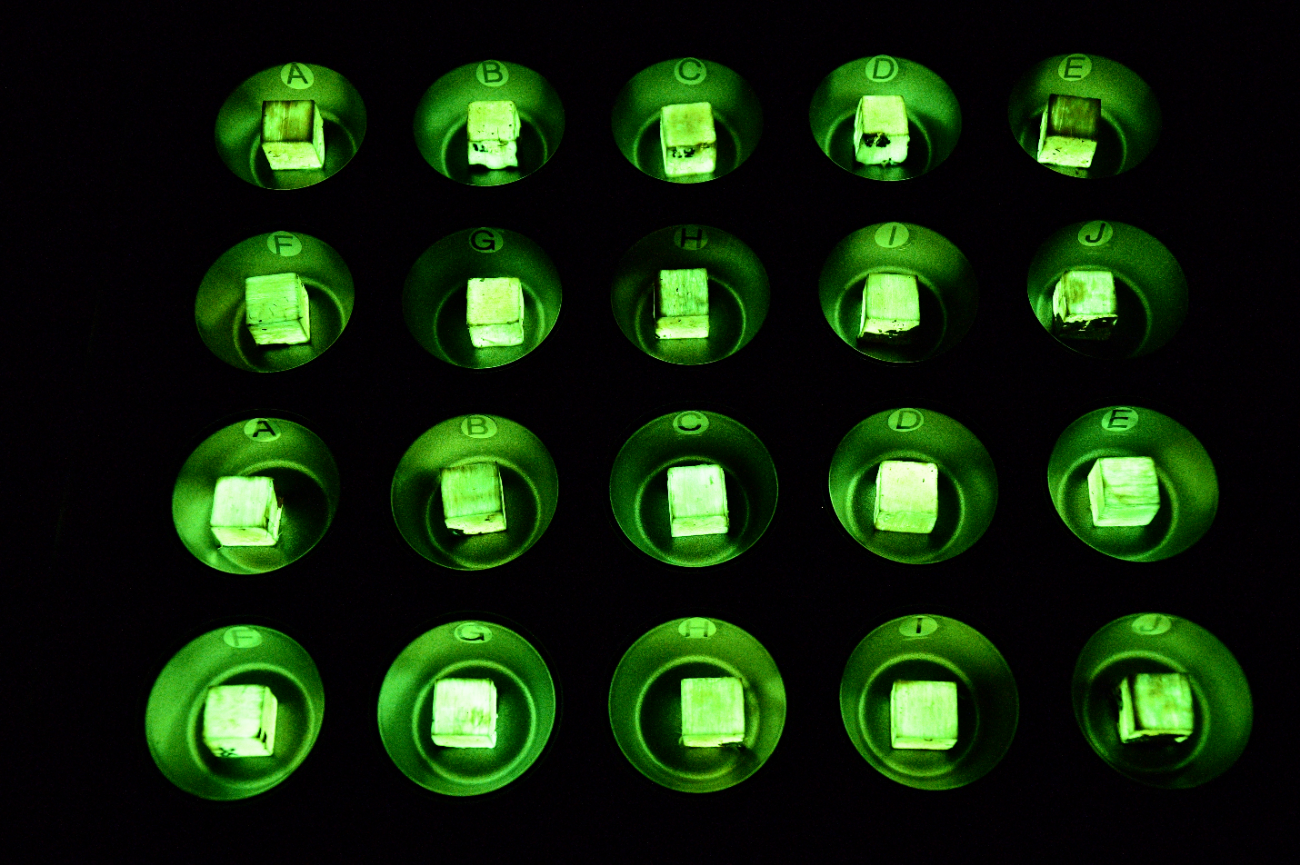
**

**Figure S 2.** Balsa wood blocks incubated for 3 months with D. tabescens made showing strong bioluminescence emission (above two rows of wood blocks without malt, below two rows with malt) after 10 hour exposure to air in the dark illuminating letters (A-J).


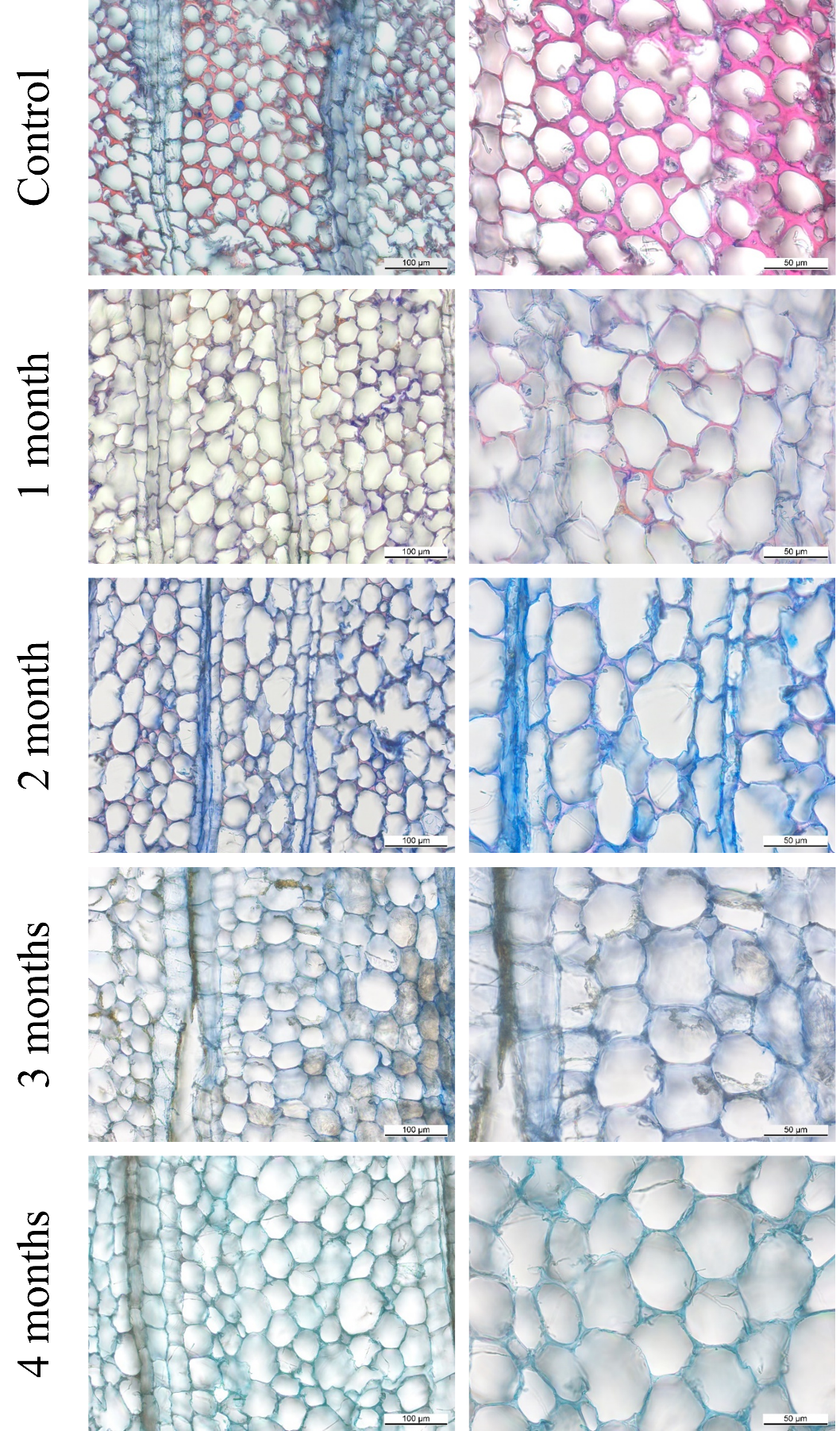


**Figure S 3.** Light microscopy imaging of the control and samples treated without malt (1-4 months), stained with Astra Blue and Safranin, to stain cellulose blue and lignin red, respectively. Scale bars on the left side micrographs are 100 µm, and the ones on the right side micrographs are 50 µm.

**Table S 2.** FTIR band areas, typical of lignin and hemicellulose, calculated through numerical analysis (Gaussian fit), of the spectra of all samples shown in **Figure 4a**. The analysis was performed using the software OriginPro 2024b.

| **Band** | **Control** | **1 month (-)** | **2 months (-)** | **3 months (-)** | **4 months (-)** | **1 month (+)** | **2 months (+)** | **3 months (+)** | **4 months (+)** |
| --- | --- | --- | --- | --- | --- | --- | --- | --- | --- |
| **1460 (Lignin)** | 12.25% | 14.15% | 4.63% | 4.32% | 5.48% | 5.96% | 6.49% | 6.46% | 5.89% |
| **1736 (Hemicellulose)** | 27.48% | 22.99% | 21.26% | 20.56% | 20.60% | 17.43% | 18.98% | 20.18% | 17.43% |

**Figure S 4.** ATR-FTIR spectra of all 9 different balsa wood samples analysed in this work.

**
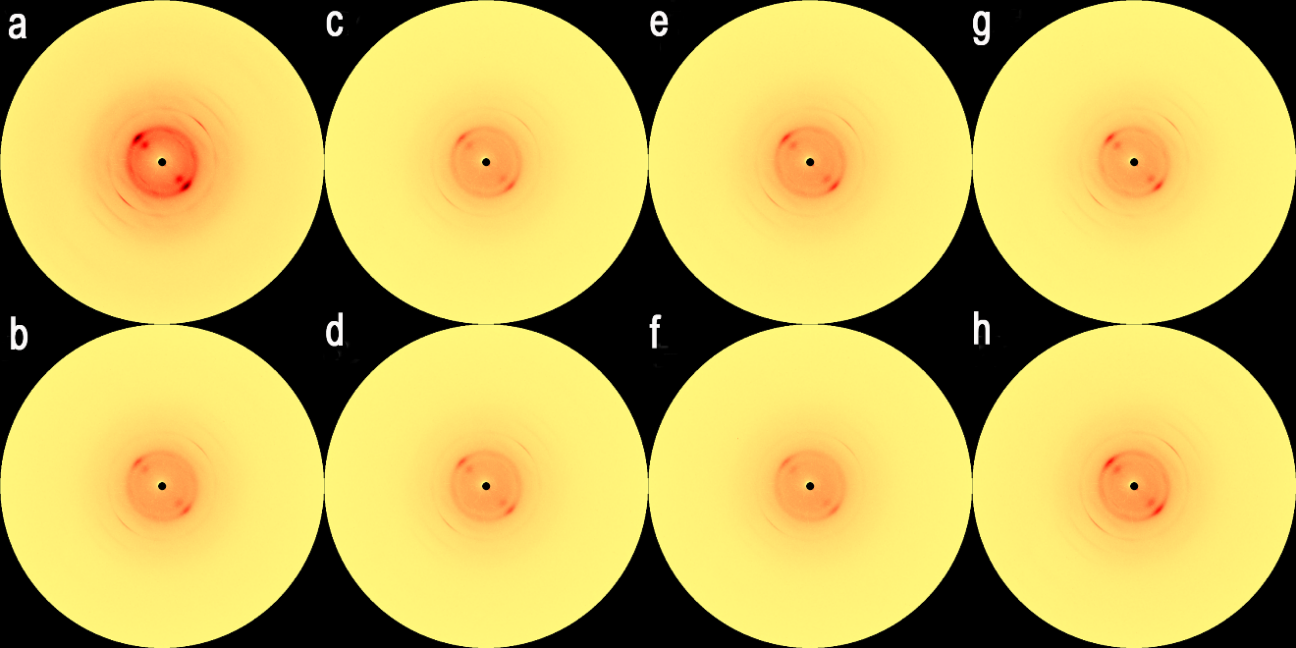
**

**Figure S 5.** 2D detector images of untreated wood showing the characteristic diffraction patterns of the I_β_ phase of cellulose. a) 1 month M+. b) 1 month M-. c) 2 months M+. d) 2 months M-. e) 3 months M+. f) 3 months M-. g) 4 months M+. h) 4 month M-.

**
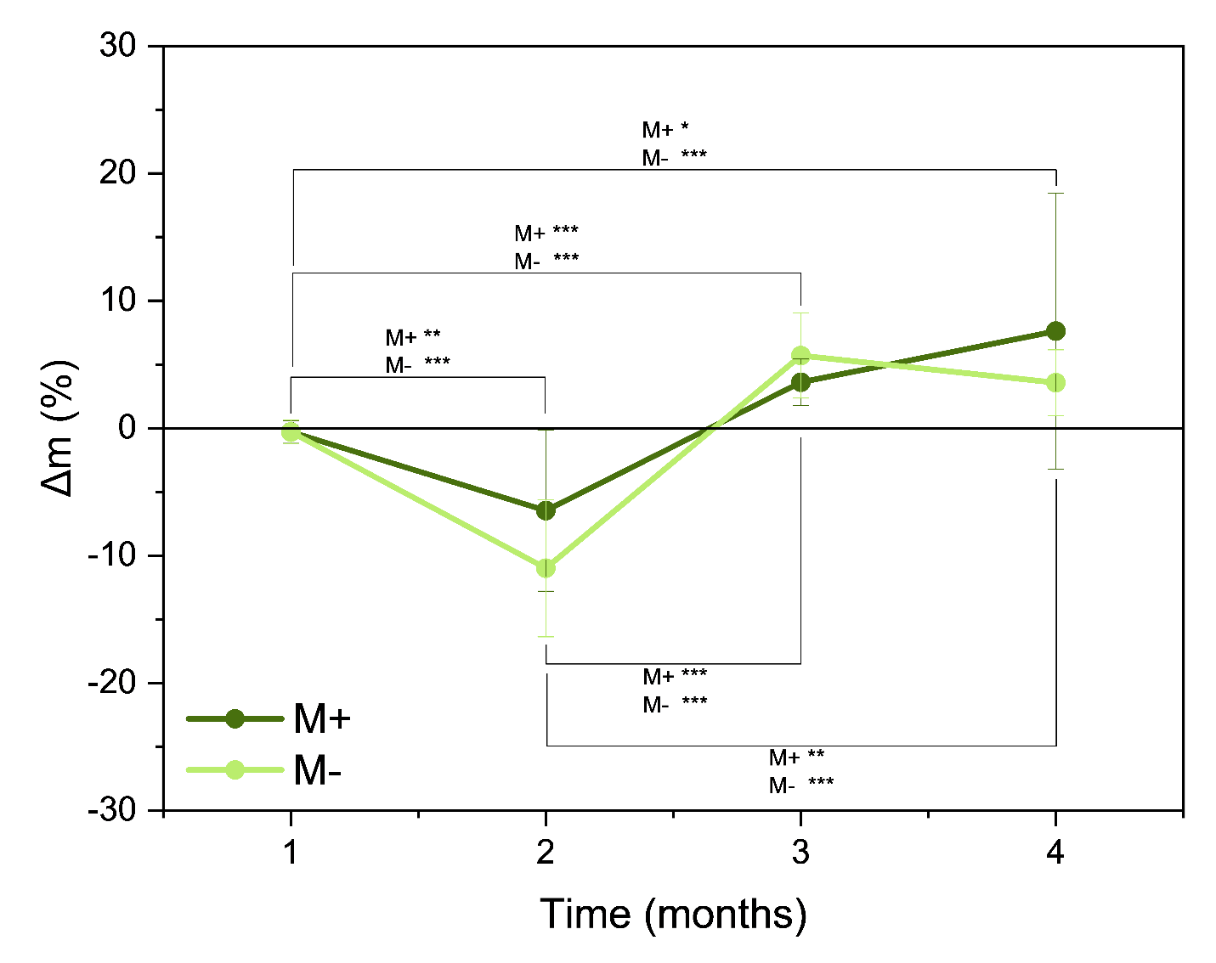
**

**Figure S 6.** Mass variation (Δm), of balsa wood blocks incubated for 1-4 months with D. tabescens, with and without addition of malt (M+ and M-, respectively). Δm shows the percentage difference between the initial dry mass and the final dry weight of the wood blocks. The results are expressed as mean ± SD (n =10). Levels of significance were set at probabilities of *p < 0.05, **p < 0.01 and ***p < 0.001 and determined by one-way ANOVA


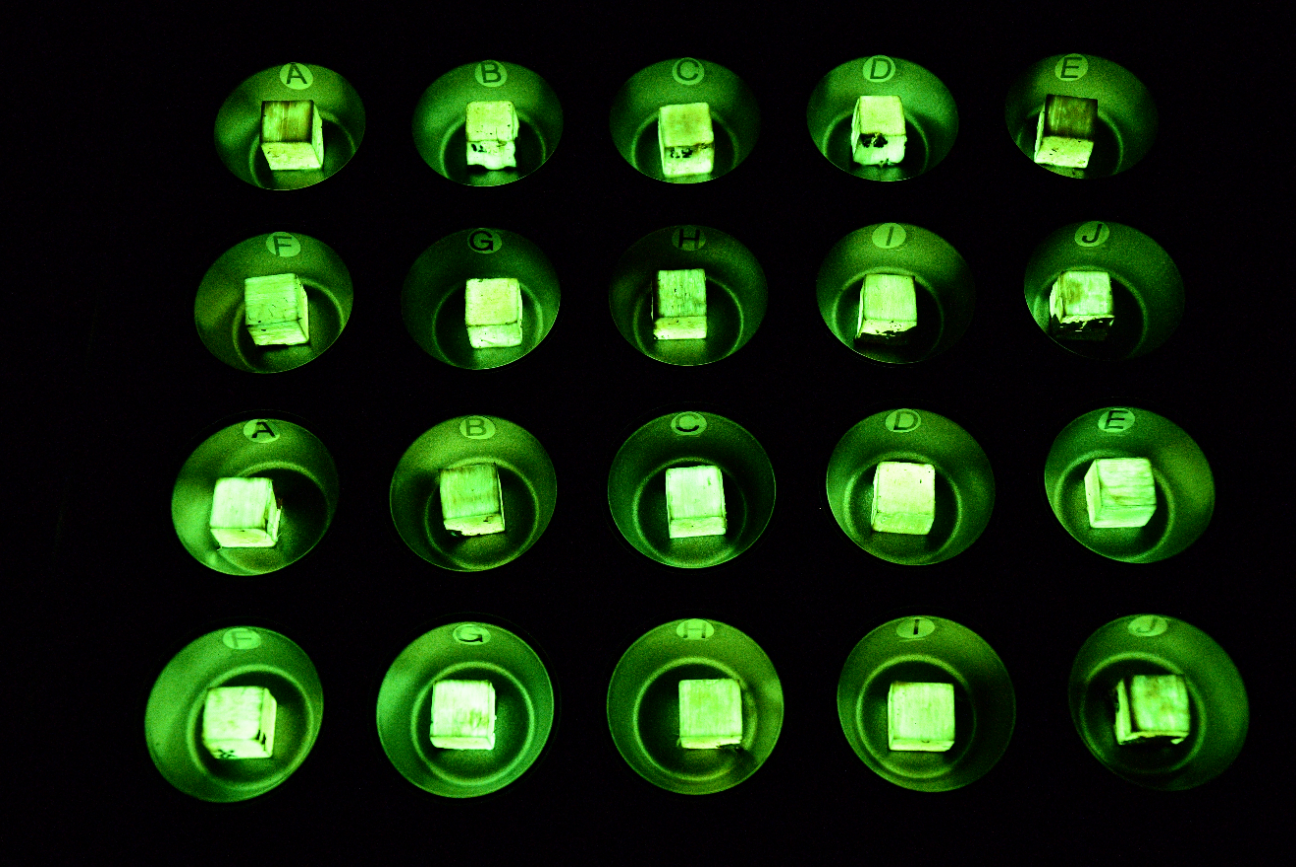


**Movie S 1.** Movie made in the dark over 65 h, showing bioluminescence emission of wood blocks (above two rows of wood blocks without malt, below with malt) and illumination of letters (A-J) after 3 months incubation with D. tabescens. Note how rehydration of wood positively influences bioluminescence after 48 h.
